# Possible Involvement of Hippocampal miR‐539‐3p/*Lrp6*/*Igf1r* Axis for Diminished Working Memory in Mice Fed a Low‐Carbohydrate and High‐Protein Diet

**DOI:** 10.1002/mnfr.202400648

**Published:** 2024-12-20

**Authors:** Takeru Shima, Hayate Onishi, Chiho Terashima

**Affiliations:** ^1^ Department of Health and Physical Education Cooperative Faculty of Education Gunma University Maebashi Gunma Japan; ^2^ Course of Biomedical Sciences in Graduate School of Medicine Gunma University Maebashi Gunma Japan

**Keywords:** carbohydrate, hippocampus, *Igf1r*, miRNA, protein, working memory

## Abstract

A low‐carbohydrate and high‐protein (LC‐HP) diet demonstrates favorable impacts on metabolic parameters, albeit it leads to a decline in hippocampal function with the decreased expression of hippocampal insulin‐like growth factor‐1 receptor (IGF‐1R) among healthy mice. However, the precise mechanisms underlying this phenomenon remain unexplored. Eight‐week‐old male C57BL/6 mice were divided into the LC‐HP diet‐fed group (25.1% carbohydrate, 57.2% protein, and 17.7% fat as percentages of calories; *n* = 10) and the control diet‐fed group (58.9% carbohydrate, 24.0% protein, and 17.1% fat; *n* = 10). After 4 weeks, all mice underwent the Y‐maze test, followed by analyses of hippocampal mRNA and miRNA expressions. We revealed that feeding the LC‐HP diet suppressed working memory function and hippocampal *Igf1r* mRNA levels in mice. Sequencing of hippocampal miRNA demonstrated 17 upregulated and 27 downregulated miRNAs in the LC‐HP diet‐fed mice. Notably, we found decreased hippocampal mRNA levels of low‐density lipoprotein receptor‐related protein 6 (*Lrp6*), a gene modulated by miR‐539‐3p, in mice fed the LC‐HP diet. Furthermore, a significant positive correlation was observed between *Lrp6* and *Igf1r* mRNA levels in the hippocampus. These findings suggest that LC‐HP diets may suppress hippocampal function via the miR‐539‐3p/*Lrp6*/*Igf1r* axis.

## Graphical abstract

We tested the effects of feeding low‐carbohydrate and high‐protein (LC‐HP) diets over a span of four weeks on hippocampal working memory in healthy mice and the alterations in hippocampal miRNA profiles to gain insight into the modulation of hippocampal function. LC‐HP diet feeding leads to a decline in working memory function with upregulation of hippocampal miR‐539‐3p and downregulation of hippocampal Igf1r and Lrp6 mRNA levels in healthy mice. These findings suggest that the LC‐HP diet suppresses hippocampal function in healthy mice through the miR‐539‐3p/Lrp6/Igf1r axis.

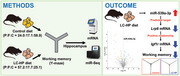

**Abbreviations**
Adamts5: a disintegrin and metalloproteinase with thrombospondin type 1 motif 5
Akap3: a‐kinase anchoring protein‐3
BDNF: brain‐derived neurotrophic factor
Dcx: doublecortin
IGF‐1R: insulin‐like growth factor‐1 receptor
LC‐HP: low‐carbohydrate and high‐protein
Lrp6: lipoprotein receptor‐related protein 6

## 1 Introduction

The nutritional composition of diets plays a pivotal role in dietary approaches aimed at preserving both physical and mental health in humans. While prior research has highlighted the advantages of low‐carbohydrate and high‐protein (LC‐HP) diets in enhancing glycemic control [1, 2], there are also documented adverse effects, including atherosclerosis and dysfunction in kidneys and vessels [3, 4, 5]. Of particular interest are the effects of such diets on brain function: while LC‐HP diets have shown benefits in obese individuals [6], they have been associated with diminished hippocampal memory function in healthy animals [7]. This observation holds significance, given the importance of maintaining hippocampal function for overall human well‐being. Consequently, unraveling the underlying mechanisms behind the LC‐HP diet‐induced decline in hippocampal function is imperative and holds considerable relevance in devising appropriate dietary strategies.

A recent investigation proposes a possible involvement of IGF‐1 receptor (IGF‐1R) with LC‐HP diets‐induced hippocampal dysfunction [7]. IGF‐1R plays a pivotal role in regulating hippocampal neurogenesis [8, 9], a critical morphological adaptation essential for maintaining hippocampal function [10, 11]. Notably, deletion of *Igf1r* gene has been shown to downregulate hippocampal neurogenesis [12]. A previous study has shown that feeding an LC‐HP diet leads to decreased hippocampal mRNA levels of *Igf1r* and *Dcx* [7], a marker of newborn immature neurons [13, 14]. Conversely, there is no observed difference in hippocampal mRNA levels of *Bdnf*, the other positive regulator of hippocampal neurogenesis [15, 16], between the mice fed an LC‐HP diet and those fed a control diet [7]. Thus, it is suggested that LC‐HP diet‐induced hippocampal dysfunction may stem from dysregulation of IGF‐1R‐related neurogenesis in the hippocampus. However, the precise mechanisms underlying the downregulation of hippocampal expressions in IGF‐1R during LC‐HP diet feeding remain to be elucidated.

The current study focused on alterations in microRNA (miRNA) expressions, which are short non‐coding RNAs typically ranging from 21 to 25 nucleotides in length [17]. MiRNAs are known to function by binding to complementary segments of messenger RNAs (mRNAs), subsequently leading to the degradation or inhibition of mRNA translation [18]. Their roles in brain development and functions have been documented [19, 20, 21], and changes in miRNA profiles are recognized as important regulators and biomarkers for dietary effects [22, 23, 24]. Moreover, variations in the nutritional composition of diets have been shown to influence miRNA profiles in various organs and circulation [25, 26, 27, 28]. Previous studies have indicated that IGF‐1R expression can be regulated by specific miRNAs, including miR‐96, miR‐181b, and miR‐223 [29, 30, 31]. Specifically, an upregulation of miR‐96 has been associated with decreased hippocampal IGF‐1R levels and consequent memory dysfunction [29]. While there is a possibility that these miRNAs may be implicated in LC‐HP diet‐induced hippocampal dysfunction and the subsequent decrease in hippocampal *Igf1r* mRNA levels, the specific changes in hippocampal miRNA profiles resulting from LC‐HP diet consumption remain unclear.

Here, we tested the effects of feeding LC‐HP diets over a span of 4 weeks on hippocampal working memory in healthy mice and the alterations in hippocampal miRNA profiles using miR‐Seq to gain insight into the modulation of hippocampal IGF‐1R expression.

## 2 Materials and Methods

### 2.1 Animals

Eight‐week‐old male C57BL/6 mice obtained from SLC Inc. (Shizuoka, Japan) were housed in a temperature‐controlled facility set at 21–23 °C and maintained on a 12‐h light/dark cycle (lights on from 7:00 to 19:00). During the acclimatization period, the mice had access to a standard pellet diet (Rodent Diet CE‐2 [58.47% carbohydrate, 29.06% protein, and 12.47% fat as percentages of calories], CLEA Japan Inc., Tokyo, Japan) and water ad libitum. The experimental procedures were pre‐approved (approval No. 23–050) and conducted in accordance with the guidelines established by the Gunma University Animal Care and Experimentation Committee.

### 2.2 Experimental Design

Following 1 week of acclimatization, the mice were divided into two groups based on matching body weights: a group receiving the LC‐HP diet (3.55 kcal/g, 25.1% carbohydrate, 57.2% protein, and 17.7% fat as percentages of calories; *n* = 10) and a group receiving the control diet (3.53 kcal/g, 58.9% carbohydrate, 24.0% protein, and 17.1% fat as percentages of calories; *n* = 10). These diets were obtained from CLEA Japan Inc. (Tokyo, Japan), and their compositions are delineated in Table 1. All mice had access to water ad libitum throughout the 4‐week experimental period. Subsequently, after the 4‐week feeding regimen, all mice underwent the Y‐maze test.

**TABLE 1:** Nutritional composition of the diets.

Ingredients	%	
	Control	LC‐HP
Cornstarch (including alpha‐cornstarch)	46.2	11.9
Casein	24.5	58.8
Sucrose	10.0	10.0
Mineral mix	7.0	7.0
Corn oil	6.0	6.0
Cellulose	3.0	3.0
Cellulose powder	2.0	2.0
Vitamin mix	1.0	1.0
Choline chloride	0.3	0.3

Abbreviation: LC‐HP, low‐carbohydrate and high‐protein diet.

### 2.3 Evaluation of Working Memory

The Y‐maze test was conducted following previously described [7]. Prior to the test, mice underwent a 3‐day acclimation period in a soundproof room for 30 min daily. Subsequently, each mouse was placed in the maze's center and allowed to freely explore the maze (with each arm measuring 43 cm in length, 4 cm in width, and 12 cm in wall height, O'hara & Co., Ltd., Japan). The sequence and frequency of entries into the arms were recorded over 8 min using a video tracking system (O'hara & Co., Ltd., Japan). An alternation was defined as consecutive navigation through all three arms without revisiting any previously entered arms. The % alternation for each mouse was calculated (% alternation = the number of alternation/[the total number of arm entries − 2] × 100).

### 2.4 Evaluation of Spatial Learning and Memory

Mice fed the control diet (*n* = 8) and those fed the LC‐HP diet (*n* = 8) for 4 weeks were subjected to the Morris water maze test. These mice were distinct from those used in the Y‐maze test for assessing working memory. The Morris water maze test was conducted in a circular pool (100 cm in diameter and 30 cm in depth) with an invisible platform (10 cm in diameter) positioned at the center of one quadrant. The experimental room included several extra‐maze cues. All four start points were employed in different sequences during the learning sessions. Mice were allotted 60 s to explore the platform. If a mouse failed to find the platform within this time, it was manually guided to it. Upon reaching the platform, mice remained there for 10 s. Throughout the learning sessions, escape latency (s), swim length (cm), and speed (cm/s) were recorded using a video tracking system (O'hara & Co., Ltd., Japan). One day following the final learning session, the platform was removed from the pool, and a probe trial was conducted, allowing the mice 60 s to search within the pool. The time spent in the quadrant where the platform had been located during the learning sessions was measured using the same tracking system.

### 2.5 Tissue Preparation

Two days after the Y‐maze test, mice were anesthetized with isoflurane (30% isoflurane in propylene glycol; Dainippon Sumitomo Pharma Co., Osaka, Japan), and the blood samples were obtained from mice by cardiac puncture. And then, the hippocampus was collected using a modified version of the method by Hirano et al. [32], and preserved in RNAlater Stabilization Solution (Invitrogen). The hippocampus samples were stored at −20 °C for subsequent biochemical analysis.

### 2.6 Blood Glucose and β‐Ketone Assay

Glucose and β‐ketone levels in the blood samples obtained from mice by cardiac puncture at the endpoint were measured by the FreeStyle Precision Neo meter (FreeStyle Libre, Abbot, Japan).

### 2.7 Real‐Time PCR

Total RNA was extracted from the hippocampus tissue using RNeasy Mini Kit with DNase I treatment (Qiagen Inc., USA) according to the protocol provided. RNA quantification was carried out using the Qubit 4.0 (Invitrogen, USA), and then 1000 ng of RNA was reverse transcribed to cDNA with GeneAce cDNA Synthesis Kit (NIPPON Genetics Co., Ltd., Japan). After that, we measured the mRNA levels of target genes using 5.0 ng of cDNA, primers for each target gene, and PowerTrack SYBR Green Master Mix in StepOne Plus Real‐Time PCR 96‐well system (Thermo Fisher Scientific Inc., USA). The sequences of primers (forward and reverse) are shown in Table S1. The relative levels of each mRNA were calculated by the ΔΔCT method and normalized by *β‐actin* mRNA levels.

### 2.8 miRNA Isolation and NGS of miRNA

We used the miRNeasy Micro Kit (Qiagen, Inc., Valencia, CA, USA) following the manufacturer's instructions for miRNA isolation from the hippocampus. The libraries for miRNA sequencing were prepared utilizing 100.0 ng of RNA, QIAseq miRNA Library Kit, and QIAseq miRNA NGS 48 Index IL (Qiagen Inc., Valencia, CA, USA), according to the manufacturer's protocol. The library quality was evaluated by measuring the library size in base pairs using the Agilent High Sensitivity DNA Kit and Agilent Bioanalyzer (Agilent Technologies, Santa Clara, CA, USA). During the library construction, each miRNA molecule was tagged with a unique molecular identifier (UMI). Single‐end sequencing of 86‐bp reads was performed on the NextSeq 500 using the NextSeq 500/550 High Output Kit v2.5 (Illumina Inc., San Diego, CA, USA). The sequenced data underwent calibration, adapter trimming, identification of insert sequencing and UMI sequencing, alignment to the mouse‐specific miRbase mature database and GRCm38 sequence, and read counting utilizing the “Primary Quantification” tool on GeneGlobe (https://geneglobe.qiagen.com/jp/). The miRDB database (https://www.mirdb.org) was utilized to predict the miRNAs targeting *Igf1r* gene and the pathway associated with IGF‐1R expression.

### 2.9 Statistical Analysis

All data are expressed as mean ± standard error (SEM) and were analyzed using Prism version 10.2.2 (MDF, Tokyo, Japan). Before the analysis for comparisons between the mice fed the control diet and the mice fed the LC‐HP diet, we checked the normality of raw data distribution using histograms. When the data were normally distributed, parametric tests (unpaired *t*‐test) were used for statistical analyses. On the other hand, if we could not confirm that data were distributed normally, non‐parametric tests (Mann–Whitney *U* test) were used for statistical analyses. Group comparisons for analyzing the results of body weight, escape latency, swim length, and speed during the learning sessions of the Morris water maze test were conducted utilizing repeated two‐way ANOVA. Correlations were analyzed by Pearson correlation. Statistical significance was set at *p* < 0.05. Log_2_ fold change in each miRNA and statistical significances were analyzed using the TCC‐iDEGES‐edgeR pipeline on R (https://www.R‐project.org/, version 4.0.3) package TCC (version 1.30.0) [33] and edgeR (version 3.32.1).

## 3 Results

### 3.1 The Effects of LC‐HP Diet on Physiological and Biochemical Variables

In mice fed the LC‐HP diet, blood glucose levels and the fat‐to‐body weight ratio were significantly lower than those fed the control diet (Table 2; *p* = 0.0027, *p* = 0.0022, respectively). Conversely, the kidney‐to‐body weight ratio in mice fed the LC‐HP diet was significantly higher than in those fed the control diet (Table 2; *p* = 0.0022). However, there were no observed differences in the body weight, the amounts of food intake, or β‐ketone levels between mice fed the LC‐HP diet and those fed the control diet (Table 2).

**TABLE 2:** The effects of LC‐HP diet on physiological and biochemical variables.

	Control	LC‐HP
Body weight (g, pre)	23.99 ± 0.34	24.07 ± 0.44
(g, endpoint)	26.67 ± 0.53	26.36 ± 0.17
Food intake (g/day)	2.82 ± 0.08	2.79 ± 0.04
Blood glucose (mg/dL)	103.32 ± 2.58	90.72 ± 2.56**
Blood β‐ketone (mM)	0.77 ± 0.06	0.80 ± 0.05
Kidney weight/Body weight (mg/g)	10.86 ± 0.28	12.20 ± 0.25**
Fat weight/Body weight (mg/g)	6.83 ± 0.35	5.20 ± 0.29**

*Note*: Data are expressed as mean ± SEM.

Abbreviation: LC‐HP, low‐carbohydrate and high‐protein diet.

**p < 0.01.

### 3.2 Changes in Working Memory and Hippocampal mRNA Levels With Feeding LC‐HP Diet

**FIGURE 1:**
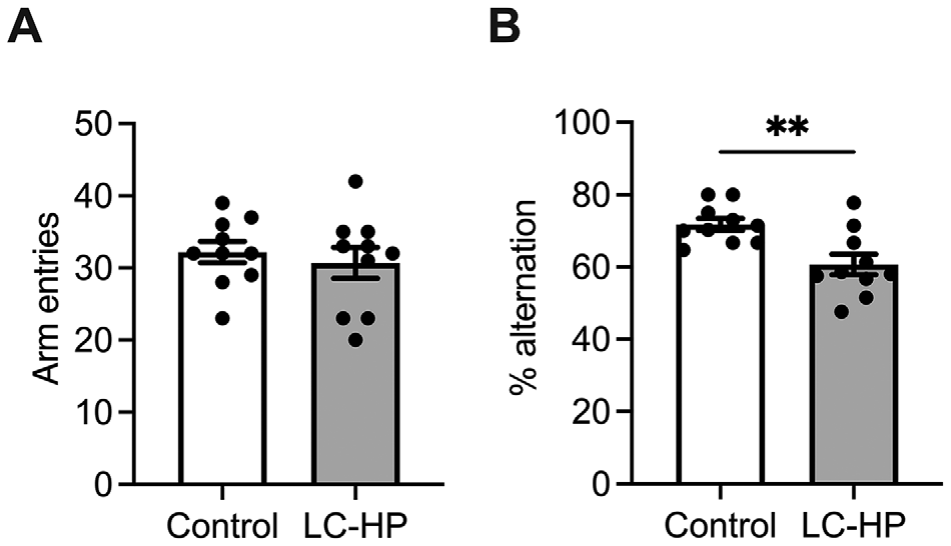
Effect of LC‐HP diet on working memory function. The number of arm entries (A), and % alternation (B) during Y‐maze. White bars: mice fed control diet, and gray bars: mice fed LC‐HP diet. Data are expressed as mean ± SEM, and analyzed by unpaired *t*‐test, *n* = 10 mice for each group. **p < 0.01. LC‐HP indicates low‐carbohydrate and high‐protein diet.

**FIGURE 2:**
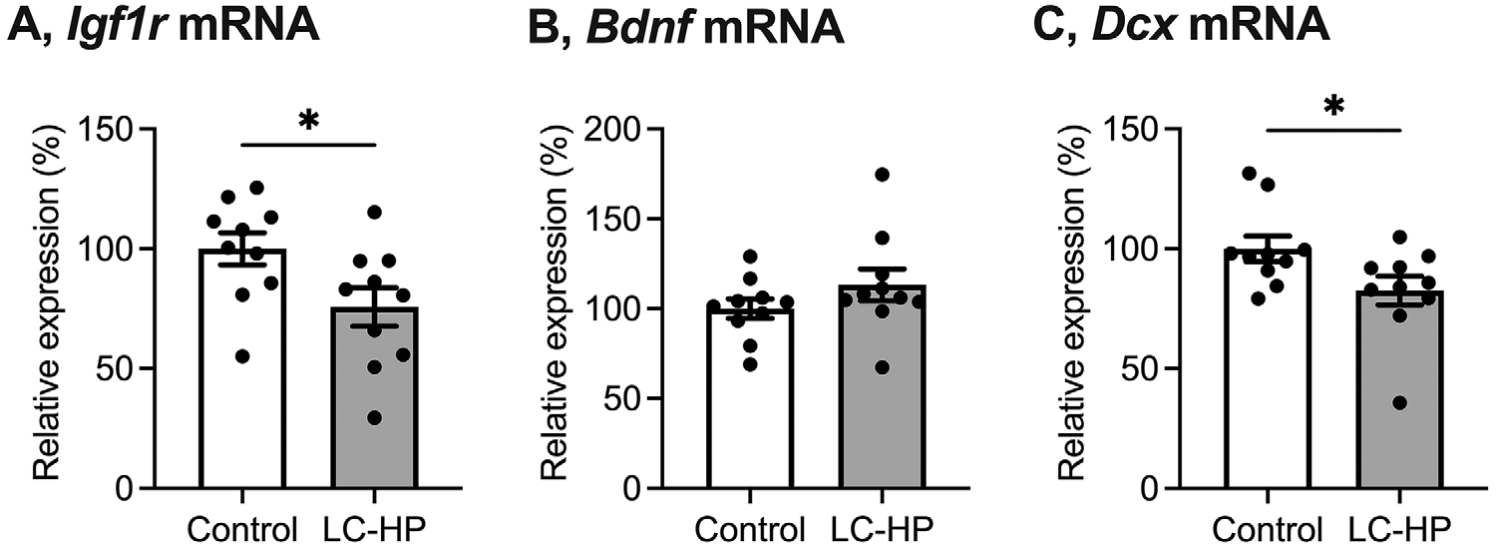
Effect of LC‐HP diet on mRNA levels of *Igf1r* (A), *Bdnf* (B), and *Dcx* (C) in the hippocampus. White bars: mice fed control diet, and gray bars: mice fed LC‐HP diet. Data are expressed as mean ± SEM, and analyzed by unpaired *t*‐test, *n* = 10 mice for each group. *p < 0.05. LC‐HP indicates low‐carbohydrate and high‐protein diet.

Although there was no difference in the number of arm entries (Figure 1A; *p* = 0.5715), mice fed the LC‐HP diet exhibited a lower % alternation in the Y‐maze test compared to those fed the control diet (Figure 1B; *p* = 0.0037), yet demonstrated no significant differences in spatial learning and memory as assessed by the Morris Water Maze (Figure S1 and Table S2). Furthermore, the mRNA levels of *Igf1r* and *Dcx* were significantly downregulated in response to the LC‐HP diet (Figure 2A,C; *p* = 0.0321, *p* = 0.0425, respectively). On the other hand, *Bdnf* mRNA levels in the hippocampus of mice remained unaltered upon feeding the LC‐HP diet (Figure 2B; *p* = 0.2170).

### 3.3 Quantification of miRNA in the Hippocampus

**FIGURE 3:**
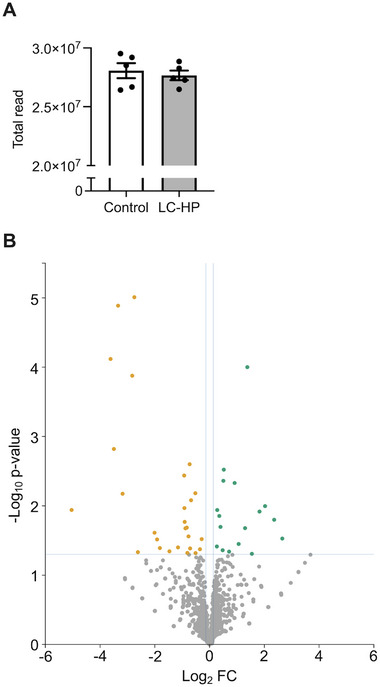
Total reads of miRNA by miR‐seq in the hippocampus for each group (A). White bars: mice fed control diet, and gray bars: mice fed LC‐HP diet. Data are expressed as mean ± SEM, and analyzed by unpaired *t*‐test, *n* = 5 mice for each group. LC‐HP indicates low‐carbohydrate and high‐protein diet. Significantly modulated miRNA in the hippocampus with feeding LC‐HP diet (B).

There was no significant difference in the total reads of hippocampal miRNA between mice fed the LC‐HP diet and those fed the control diet (Figure 3A; *p* = 0.6129). Through miRNA sequencing, it was found that 44 kinds of hippocampal miRNA were significantly regulated by feeding LC‐HP diet; among these, 17 were upregulated and 27 were downregulated (Figure 3B). The fold changes for these miRNAs are provided in Tables S3 and S4. In addition, 6 out of 17 upregulated miRNAs and 4 out of 27 downregulated miRNAs showed a significant correlation with % alternation in the Y‐maze test (Tables S3 and S4). Notably, among the six miRNAs upregulated by the LC‐HP diet and associated with working memory, miR‐539‐3p, miR‐743a‐3p, and miR‐3086‐3p are predicted to target the *Igf1r* gene, as per miRDB (https://www.mirdb.org).

### 3.4 Changes in miR‐539‐3p‐Related mRNA Levels in the Hippocampus With Feeding LC‐HP Diets

**FIGURE 4:**
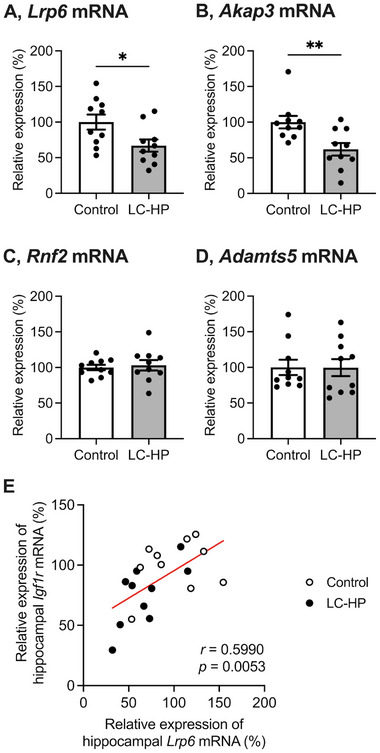
Effect of LC‐HP diet on mRNA levels of *Lrp6* (A), *Akap3* (B), *Rnf2* (C), and *Adamts5* (D) in the hippocampus. White bars: mice fed control diet, and gray bars: mice fed LC‐HP diet. Data are expressed as mean ± SEM, and analyzed by unpaired *t*‐test, *n* = 10 mice for each group. *p < 0.05, **p < 0.01. LC‐HP indicates low‐carbohydrate and high‐protein diet. The correlations between mRNA levels of *Lrp6* and *Igf1r* in the hippocampus (E) were analyzed by Pearson correlation. The line in the scatter diagram indicates significant correlation.

The mice fed the LC‐HP diet exhibited significantly lower hippocampal mRNA levels of low‐density lipoprotein receptor‐related protein 6 (*Lrp6*), a gene regulated by miR‐539‐3p, compared to those fed the control diet (Figure 4A; *p* = 0.0261). In addition, hippocampal *Lrp6* mRNA levels showed a significant positive correlation with hippocampal *Igf1r* mRNA levels (Figure 4E; *r* = 0.5990, *p* = 0.0053). Regarding other mRNAs regulated by miR‐539‐3p, hippocampal mRNA levels of a‐kinase anchoring protein‐3 (*Akap3*) were decreased with feeding the LC‐HP diet, whereas no significant changes were observed in the mRNA levels of ring finger protein 2 (*Rnf2*) or a disintegrin and metalloproteinase with thrombospondin type 1 motif 5 (*Adamts5*) (Figure 4B–D; *p* = 0.0067, *p* = 0.7081, *p* = 0.9829, respectively).

## 4 Discussion

The current study investigated the effects of the LC‐HP diet on hippocampal working memory and the levels of miRNA and mRNA in the hippocampus. Our findings reaffirm that feeding the LC‐HP diet leads to a reduction in the % alternation in the Y‐maze, accompanied by decreased hippocampal *Igf1r* and *Dcx* mRNA levels. Furthermore, we observed significantly elevated levels of miR‐539‐3p and reduced levels of *Lrp6* mRNA in the hippocampus of mice fed the LC‐HP diet compared to those fed the control diet.

The current study, consistent with previous research [2–4, 7], found that the LC‐HP diet induced alterations in physiological and biochemical variables in mice (Table 2). Further, the mice fed the current LC‐HP diet did not exhibit nutritional ketosis. A previous study reported that mice fed a ketogenic diet (10% protein, <1% carbohydrate, and 89% fat) showed elevated circulating beta‐hydroxybutyrate levels compared to mice fed a control diet (18% protein, 65% carbohydrate, and 17% fat). However, mice fed a low‐carbohydrate diet (20% protein, 10% carbohydrate, and 70% fat) did not show such an increase [34]. Thus, the lack of change in circulating ketone body levels in mice on the current LC‐HP diet is understandable. After 4 weeks on the LC‐HP diet, healthy mice exhibited significant impairment in working memory (Figure 1B) and a reduction in hippocampal mRNA levels of *Igf1r* and *Dcx* (Figure 2A,C). These results, consistent with those of a previous report [7], confirm the validity of the LC‐HP diet utilized in our study. Given the crucial role of IGF‐1R in regulating neurogenesis [12], it is assumed that IGF‐1R‐related neurogenesis in the hippocampus is involved in LC‐HP diet‐induced suppression of working memory based on our findings and prior reports [7]. Nevertheless, our study did not investigate morphological changes in the hippocampus associated with LC‐HP diet feeding, indicating the necessity for further mechanism‐based investigations.

Based on the miR‐Seq results in our study, we identified three miRNAs (miR‐539‐3p, miR‐743a‐3p, and miR‐3086‐3p) predicted to target *Igf1r* gene according to miRDB (https://www.mirdb.org) among the upregulated hippocampal miRNAs associated with LC‐HP diet feeding and related to working memory. Of particular interest was miR‐539‐3p, as it is also predicted to target pathways related to IGF‐1R. Previous research has demonstrated that miR‐539‐3p downregulates *Lrp6* mRNA levels [35], and LRP6, in turn, regulates expressions and activation of IGF‐1R [36, 37]. LRP6 plays a critical role in Wnt/β‐catenin signal transduction [38, 39, 40]. Further, LRP6/Wnt/β‐catenin/IGF‐1R signaling would contribute to cell growth [37]. Importantly, our study revealed a significant positive correlation between *Lrp6* and *Igf1r* mRNA in the hippocampus (Figure 4E). Therefore, there is a possibility that the LC‐HP diet suppresses hippocampal function mediated by the miR‐539‐3p/*Lrp6*/*Igf1r* axis in healthy mice.

MiR‐539‐3p targets not only *Igf1r* and *Lrp6* genes but also *Akap3*, *Rnf2*, and *Adamts5* genes, as reported in previous studies [35, 41, 42]. Our current study revealed that feeding the LC‐HP diet downregulated hippocampal mRNA levels of *Akap3*, while no significant changes were observed in *Rnf2* or *Adamts5* mRNAs (Figure 4B–D). AKAPs bind protein kinase A (PKA) and enhance neuroplasticity [43, 44, 45]. Therefore, the suppression of hippocampal function in healthy mice fed the LC‐HP diet could be mediated by both the miR‐539‐3p/*Lrp6*/*Igf1r* axis and the miR‐539‐3p/*Akap*3 axis. However, our study did not investigate this aspect, indicating the need for further research in the future. Furthermore, miR‐539‐3p is also predicted to target *Bdnf* gene according to miRDB (https://www.mirdb.org). However, consistent with a previous report [7] and our current finding (Figure 2B), hippocampal *Bdnf* mRNA levels remained unaltered when feeding the LC‐HP diet. These findings suggest that miR‐539‐3p primarily targets *Igf1r*, *Lrp6*, and *Akap3* genes rather than *Rnf2*, *Adamts5*, and *Bdnf* genes in the mice fed the LC‐HP diet.

Several miRNAs showing significant alterations in miR‐Seq in our current study (Figure 3B and Tables S3 and S4) have been implicated in neuroplasticity. For instance, upregulation of hippocampal miR144‐3p has been observed in rodent models of schizophrenia [46], which is characterized by working memory deficit as a complication [47]. Additionally, a deficiency of miR‐298‐5p is associated with neurotoxicity [48]. Our results indicated increased levels of miR144‐3p and decreased levels of miR‐298‐5p in the hippocampus of mice fed LC‐HP diet (Figure 3B and Table S4), suggesting the involvement of these miRNAs in LC‐HP diet‐induced suppression of hippocampal function. Conversely, mice fed the LC‐HP diet exhibited several changes in hippocampal miRNAs that could improve hippocampal function. Previous studies have reported that downregulation of miR‐200c‐3p and miR‐335‐3p contribute to improving hippocampal apoptosis and cognitive dysfunction [49, 50]. However, in our study, downregulation of these miRNAs and suppression of working memory were concurrently observed in mice fed the LC‐HP diet (Figure 3B and Table S4). Therefore, further mechanism‐based investigations are warranted to elucidate the crucial pathways involved in LC‐HP diet‐induced suppression of hippocampal function.

The current study has some limitations. Firstly, we utilized only one specific composition of the LC‐HP diet. Given that the sources of nutritional ingredients can influence the cognitive effects of dietary interventions [51, 52], further validation is necessary. Secondly, our study focused solely on regulating hippocampal *Igf1r* mRNA, which does not conclusively establish the importance of hippocampal IGF‐1R in LC‐HP diet‐induced suppression of working memory. Future studies should investigate the effects of feeding the LC‐HP diet in conjunction with pharmacological activation and genetic overexpression of hippocampal IGF‐1R. Thirdly, we did not assess hippocampal protein levels or morphological changes in our study, which could provide valuable insights. Fourthly, our evaluation was confined to adaptations within the hippocampus. It is known that peripheral tissue‐derived miRNAs packaged in exosomes can regulate hippocampal plasticity and function [53, 54]. Therefore, peripheral organ‐derived miRNAs may influence alterations in hippocampal miRNA profiles observed in our study. Exploring the crosstalk between peripheral organs and the hippocampus necessitates further investigation. Fifthly, the current study measured mRNA and miRNA levels in the entire hippocampus of the mice. Previous research has highlighted the significance of the CA1 and CA3 regions in working memory performance [55, 56]. Thus, future studies should examine changes in molecular levels within each hippocampal subregion. Finally, it is essential to investigate whether the current findings in animal models can be translated to human conditions in future research.

In conclusion, our study demonstrates that 4 weeks of LC‐HP diet feeding leads to a decline in working memory function, accompanied by upregulation of hippocampal miR‐539‐3p and downregulation of hippocampal *Igf1r* and *Lrp6* mRNA levels in healthy mice. These findings suggest that the LC‐HP diet suppresses hippocampal function in healthy mice through the miR‐539‐3p/*Lrp6*/*Igf1r* axis, indicating potential new targets for innovating and developing nutritional strategies to maintain and enhance hippocampal function.

## Conflicts of Interest

The authors declare no conflicts of interest.

## Supporting information



Supporting Information

## Data Availability

The datasets in the current study are available from the corresponding author on reasonable request.
