# Supplementary material for: Possible Involvement of Hippocampal miR‐539‐3p/Lrp6/Igf1r Axis for Diminished Working Memory in Mice Fed a Low‐Carbohydrate and High‐Protein Diet
Source: Mol Nutr Food Res. 2024 Dec 20;69(2):e202400648. doi: 10.1002/mnfr.202400648 (PMC11744036; doi:10.1002/mnfr.202400648)
Supplement: Supplementary file 1 — Supporting Information [file MNFR-69-e202400648-s001.docx]

**Supplementary Tables**

Supplementary Table 1. ﻿Primer sequences

| Gene | Primer | Sequence |
| --- | --- | --- |
| *Igf1r* | F | CCAGGGCTTGTCCAACGAGCA |
|  | R | TCCCGGAAGCCAGGCTCCAT |
| *Bdnf* | F | GATGAGGACCAGAAGGTTCG |
|  | R | GATTGGGTAGTTCGGCATTG |
| *Trkb* | F | ATCCTGGTGGCCGTGAAG |
|  | R | TGAAAGTCCTTGCGAGCATTG |
| *Creb1* | F | TCAGCCGGGTACTACCATTC |
|  | R | TCTCTTGCTGCTTCCCTGTT |
| *Dcx* | F | GAGTGCGCTACATTTATACCATTG |
|  | R | TGACATTCTTGGTGTACTCAACCT |
| *Lrp6* | F | CATGGACATCCAAGTGCTGA |
|  | R | TTGTCCTCCTCGCATGGT |
| *Akap3* | F | AACAGAAAATTACTAAGCACCAACG |
|  | R | TTGGTGTCTTCACTATCCCTAAGTC |
| *Rnf2* | F | ATGCCCTACCTGTCGGAAAA |
|  | R | TCTTCAGCCCCTCCTCAATG |
| *Adamts5* | F | GCATCCAAGCCCTGGTCCAAAT |
|  | R | GGTGGCATCGTAGGTCTGTCCT |
| *β-actin* | F | TATGCCAACACAGTGCTGTCTGG |
|  | R | TACTCCTGCTTGCTGATCCACAT |

Supplementary Table 2. ﻿The effects of LC-HP diet on physiological and biochemical variables in the mice for the assessment of spatial learning and memory

|  | Control | | |  | LC-HP | | |
| --- | --- | --- | --- | --- | --- | --- | --- |
| Body weight (g, pre) | 24.68 | ± | 0.30 |  | 24.98 | ± | 0.32 |
| (g, endpoint) | 27.54 | ± | 0.25 |  | 26.83 | ± | 0.41 |
| Food intake (g/day) | 2.78 | ± | 0.09 |  | 2.80 | ± | 0.09 |
| Blood glucose (mg/dL) | 119.00 | ± | 7.16 |  | 91.75 | ± | 6.45* |
| Kidney weight/Body weight (mg/g) | 12.17 | ± | 0.42 |  | 13.69 | ± | 0.38* |
| Fat weight/Body weight (mg/g) | 7.31 | ± | 0.76 |  | 5.15 | ± | 0.40* |

Data are expressed as mean ± SEM, **p* < 0.05. LC-HP: Low-carbohydrate and high-protein diet.

Supplementary Table 3. Fold change of significant upregulated miRNA in the hippocampus associated with LC-HP diet feeding and their correlation with working memory

| Name | Hippocampal levels | |  | UMI count vs working memory | |
| --- | --- | --- | --- | --- | --- |
|  | Fold change | P-value |  | *r* | P-value |
| mmu-miR-467e-3p | 6.306 | 0.02966 |  | -0.4058 | 0.2446 |
| mmu-miR-7000-3p | 5.133 | 0.01590 |  | -0.6906 | 0.0270 |
| mmu-miR-7046-3p | 4.074 | 0.01009 |  | -0.5666 | 0.0876 |
| mmu-miR-3086-3p | 3.541 | 0.01210 |  | -0.8165 | 0.0039 |
| mmu-miR-743a-3p | 2.918 | 0.04918 |  | -0.9299 | <0.0001 |
| mmu-miR-539-3p | 2.600 | 0.00010 |  | -0.7862 | 0.0070 |
| mmu-miR-144-3p | 2.456 | 0.02097 |  | -0.5255 | 0.1188 |
| mmu-miR-6970-3p | 2.088 | 0.03552 |  | -0.6559 | 0.0394 |
| mmu-miR-144-5p | 1.886 | 0.00468 |  | -0.6363 | 0.0479 |
| mmu-miR-7021-5p | 1.640 | 0.04575 |  | -0.4364 | 0.2074 |
| mmu-miR-497a-3p | 1.433 | 0.00301 |  | -0.4124 | 0.2362 |
| mmu-miR-19b-3p | 1.417 | 0.00436 |  | -0.1943 | 0.5907 |
| mmu-miR-3101-5p | 1.392 | 0.04371 |  | -0.3998 | 0.2523 |
| mmu-miR-3069-3p | 1.318 | 0.02013 |  | -0.5625 | 0.0905 |
| mmu-miR-101a-3p | 1.279 | 0.01400 |  | -0.2335 | 0.5161 |
| mmu-miR-551b-3p | 1.214 | 0.01150 |  | -0.2125 | 0.5556 |
| mmu-miR-218-2-3p | 1.202 | 0.03857 |  | -0.1396 | 0.7004 |

Supplementary Table 4. Fold change of significant downregulated miRNA in the hippocampus associated with LC-HP diet feeding and their correlation with working memory

| Name | Hippocampal levels | |  | UMI count vs working memory | |
| --- | --- | --- | --- | --- | --- |
|  | Fold change | P-value |  | *r* | P-value |
| mmu-miR-216b-3p | -32.910 | 0.01148 |  | 0.1094 | 0.7635 |
| mmu-miR-216a-3p | -12.270 | 0.00008 |  | 0.0597 | 0.8698 |
| mmu-miR-217-3p | -11.279 | 0.00151 |  | 0.0659 | 0.8565 |
| mmu-miR-217-5p | -10.142 | 0.00001 |  | 0.0596 | 0.8701 |
| mmu-miR-489-3p | -9.054 | 0.00670 |  | 0.0511 | 0.8886 |
| mmu-miR-216a-5p | -7.087 | 0.00013 |  | 0.0369 | 0.9193 |
| mmu-miR-3547-3p | -6.716 | 0.00001 |  | 0.0562 | 0.8775 |
| mmu-miR-344e-5p | -6.144 | 0.04652 |  | 0.0774 | 0.8316 |
| mmu-miR-1247-5p | -4.027 | 0.02444 |  | 0.3485 | 0.3236 |
| mmu-miR-365-1-5p | -3.777 | 0.03055 |  | 0.4638 | 0.1770 |
| mmu-miR-7662-3p | -3.519 | 0.04065 |  | 0.6702 | 0.0339 |
| mmu-miR-7032-3p | -2.764 | 0.04533 |  | 0.6992 | 0.0244 |
| mmu-miR-486a-3p | -2.225 | 0.03978 |  | 0.0318 | 0.9305 |
| mmu-miR-205-5p | -1.902 | 0.00366 |  | 0.5555 | 0.0955 |
| mmu-miR-135a-1-3p | -1.890 | 0.01078 |  | 0.1722 | 0.6344 |
| mmu-miR-539-5p | -1.878 | 0.01704 |  | 0.5467 | 0.1020 |
| mmu-miR-298-5p | -1.842 | 0.02110 |  | 0.0489 | 0.8932 |
| mmu-miR-1955-3p | -1.781 | 0.02061 |  | 0.7062 | 0.0225 |
| mmu-miR-219b-5p | -1.754 | 0.04773 |  | 0.4068 | 0.2434 |
| mmu-miR-6540-3p | -1.705 | 0.02759 |  | 0.4900 | 0.1506 |
| mmu-miR-335-3p | -1.657 | 0.00251 |  | 0.2185 | 0.5442 |
| mmu-miR-200c-3p | -1.637 | 0.04115 |  | 0.1539 | 0.6712 |
| mmu-miR-490-5p | -1.598 | 0.00831 |  | 0.1865 | 0.6060 |
| mmu-miR-770-3p | -1.434 | 0.00658 |  | 0.1704 | 0.6378 |
| mmu-miR-223-3p | -1.427 | 0.04800 |  | 0.6735 | 0.0328 |
| mmu-miR-3572-3p | -1.276 | 0.04227 |  | 0.5106 | 0.1315 |
| mmu-miR-540-3p | -1.222 | 0.03022 |  | 0.3482 | 0.3242 |

**Supplementary Figure**

Supplementary Fig. 1. Effects of LC-HP diet on spatial learning and memory function. Escape latency (A), swim length (B), and speed (C) during the learning session in mice (mean ± SEM). White circles: C57BL/6 mice fed control diet, black circles: C57BL/6 mice fed LC-HP diet. White circles: C57BL/6 mice fed control diet, black circles: C57BL/6 mice fed LC-HP diet. Data are expressed as mean ± SEM, and analyzed by repeated two-way ANOVA. (D) The effect of LC-HP diet on the probe trial, showing the time spent in the platform area. White bar: C57BL/6 mice fed control diet, gray bar: C57BL/6 mice fed LC-HP diet. Data are expressed as mean ± SEM, and analyzed by unpaired t-test, n = 8/group.
